# A Comparative Survey of Methods for Remote Heart Rate Detection From Frontal Face Videos

**DOI:** 10.3389/fbioe.2018.00033

**Published:** 2018-05-01

**Authors:** Chen Wang, Thierry Pun, Guillaume Chanel

**Affiliations:** ^1^Computer Vision and Multimedia Laboratory, Computer Science Department, University of Geneva, Geneva, Switzerland; ^2^Swiss Center for Affective Sciences, Campus Biotech, University of Geneva, Geneva, Switzerland

**Keywords:** heart rate, remote sensing, physiological signals, photoplethysmography, human–computer interaction

## Abstract

Remotely measuring physiological activity can provide substantial benefits for both the medical and the affective computing applications. Recent research has proposed different methodologies for the unobtrusive detection of heart rate (HR) using human face recordings. These methods are based on subtle color changes or motions of the face due to cardiovascular activities, which are invisible to human eyes but can be captured by digital cameras. Several approaches have been proposed such as signal processing and machine learning. However, these methods are compared with different datasets, and there is consequently no consensus on method performance. In this article, we describe and evaluate several methods defined in literature, from 2008 until present day, for the remote detection of HR using human face recordings. The general HR processing pipeline is divided into three stages: face video processing, face blood volume pulse (BVP) signal extraction, and HR computation. Approaches presented in the paper are classified and grouped according to each stage. At each stage, algorithms are analyzed and compared based on their performance using the public database MAHNOB-HCI. Results found in this article are limited on MAHNOB-HCI dataset. Results show that extracted face skin area contains more BVP information. Blind source separation and peak detection methods are more robust with head motions for estimating HR.

## Introduction

Heart rate (HR) is a measure of physiological activity and it can indicate a person’s health and affective status (Malik, 1996; Armony and Vuilleumier, 2013). Physical exercise, mental stress, and medicines all influence on cardiac activities. Consequently, HR information can be used in a wide range of applications, such as medical diagnosis, fitness assessment, and emotion recognition. Traditional methods of measuring HR rely on electronic or optical sensors. The majority of these methods require skin-contact, such as electrocardiograms (ECGs), sphygmomanometry and pulse oximetry, and the later giving a photoplethysmogram (PPG). Among all cardiac pulse measurements, the current gold standard is the usage of ECG (Dawson et al., 2010), which places adhesive gel electrodes on the participants’ limbs or chest surface. Another, widely applied contact method, is to compute the blood volume pulse (BVP) from a PPG captured by an oximeter emitting and measuring light at proper wavelengths (Allen, 2007). However, the skin-contact measurements can be considered as inconvenient, unpractical, and may cause uncomfortable feelings.

In the past decade, researchers have focused on remote (i.e., contactless) detection methods, which are mainly based on computer vision techniques. Using human faces as physiological measurement resources was first proposed in 2007 (Pavlidis et al., 2007). According to Pavlidis et al. (2007), the face area facilitated observation as it featured a thin layer of tissue. With facial thermal imaging, HR can be detected based on bioheat models (Garbey et al., 2007; Pavlidis et al., 2007). After that, the PPG technique, which is non-invasive and optical, was used for detecting HR. The method is often implemented with dedicated light sources such as red lights or infrared lights (Allen, 2007; Jeanne et al., 2013).

In 2008, Verkruysse et al. (2008) showed the possibility of using PPG under ambient light to estimate HR from videos of human face. Then in 2010, Poh et al. (2010) developed a framework for automatic HR detection using the color of human face recordings obtained from a standard camera. This framework was widely adopted and modified in Poh et al. (2011), Pursche et al. (2012), and Kwon et al. (2012). For all those methods, the core idea is to recover the heartbeat signal using blind source separation (BSS) on the temporal changes of face color. Later in 2013, another method for estimating HR based on subtle head motions (Balakrishnan et al., 2013, Rubinstein, 2013) was proposed. Besides, researchers (Li et al., 2014; Stricker et al., 2014; Xu et al., 2014) investigated the estimation of HR directly by applying diverging noise reduction algorithms and optical modeling methods. Alternatively, the usage of manifold learning methods mapping multi-dimensional face video data into one-dimensional space has been studied to reveal the HR signal as well.

As shown above, remote HR detection has been an active field of research for the past decade and produced different strategies using diverse processing methods and models. However, many implementations were evaluated on different datasets and it is consequently difficult to compare them. Furthermore, no survey paper has been conducted, with the objective of gathering, classifying, and analyzing the existing work within this domain. The objectives of this article are first to fill this gap by presenting a general pipeline composed of several steps and how the different state-of-the-art methods can be classified based on the pipeline (presenting in Section “Remote Methods for HR Detection”). The second objective is to evaluate the mainstream methods at each step of the pipeline to finally obtain a full implementation with the best performance (presenting in Section “Comparative Analysis”). This objective is achieved by testing the methods on a unique set of data: the MAHNOB-HCI database. Given the methods’ popularity, this analysis is limited to color intensity-based methods.

## Remote Methods for HR Detection

To the best of our knowledge, we are unaware of existing reviews that touch upon this topic. To access the method performance, this article investigates several methods, which were published in international conferences and journals from 2008 until 2017. This time period was selected because 2008 was the year when the remote HR detection was first proposed. Methods that require no skin-contact and no specific light sources were exclusively taken into account because they are more likely to be applied outside the laboratory.

**Figure 1:**
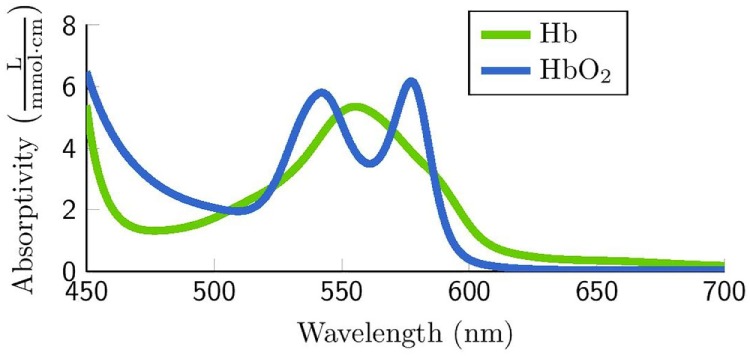
Hemoglobin (green) and oxyhemoglobin (blue) absorption spectra (Jensen and Hannemose, 2014).

The existing remote methods for obtaining HR from face videos can be classified as either color intensity-based methods or motion-based methods. Currently, the intensity-based methods are the most popular (Poh et al., 2011; Kwon et al., 2012; Pursche et al., 2012; etc.) shown in Table 1. Intensity-based methods come from PPG signals captured by digital cameras. Blood absorbs light more than the surrounding tissues and variations in blood volume affect light transmission and reflectance (Verkruysse et al., 2008). That leads to the subtle color changes on human skin, which is invisible to human eyes but recorded by cameras. Diverse optical models are applied to extract the intensity of color changes caused by pulse. As shown in Figure 1, hemoglobin and oxyhemoglobin both have high ability of absorption in the green color range and low in the red color range. But all three color channels contain PPG information (Verkruysse et al., 2008). More detailed information on PPG-based methods can be found in the work of Allen (2007) and Sun et al. (2012).

**Table 1:** Classification of state-of-the-art methods.

Dimensionality reduction	Blind source separation	Independent component analysis	Poh et al. (2010), Poh et al. (2011), Pursche et al. (2012), Kwon et al. (2012), Lewandowska et al. (2011), Sahindrakar et al. (2011), Datcu et al. (2013), Jensen and Hannemose (2014), Yu et al. (2015), Lam and Yoshinori (2015), Kumar et al. (2015), and McDuff et al. (2017)

		Principle component analysis	Lewandowska et al. (2011), Wei et al. (2012), Rubinstein (2013), Irani et al. (2014), Balakrishnan et al. (2013), and Chen et al. (2017)

	Other dimensionality methods		Wei et al. (2012), Rubinstein (2013), and Tran et al. (2015)

Optical modeling	Green channel		Verkruysse et al. (2008), Pursche et al. (2012), Stricker et al. (2014), Li et al. (2014), Zaunseder et al. (2014), Muender et al. (2016), Mestha et al. (2014), Kumar et al. (2015), and Moreno et al. (2015)

	Other optical modeling methods		Pursche et al. (2012), Stricker et al. (2014), Li et al. (2014), Zaunseder et al. (2014), Muender et al. (2016), Mestha et al. (2014), Kumar et al. (2015), and Moreno et al. (2015)

Motion-based methods			Balakrishnan et al. (2013), Rubinstein (2013), and Irani et al. (2014)

Machine learning			Monkaresi et al. (2014), Tarassenko et al. (2014), Osman et al. (2015), and Villarroel et al. (2017)

Head motions caused by pulse are mixed together with other involuntary and voluntary head movements. Subtle upright head motions in the vertical direction are mainly caused by pulse activities, while the bobbing movements are caused by respiration (Da et al., 2011; Balakrishnan et al., 2013). Motion-based methods for detecting HR stemmed from ballistocardiogram (Starr et al., 1939). Ballistocardiographic head movement is obtained by lying a participant on a low-friction platform from which displacements are measured to get cardiac information. In Da et al. (2011), head motion was measured by accelerometers to monitor HR. Balakrishnan et al. (2013) proposed to detect HR remotely from face videos through head motions. The basic approach consists of tracking features from a person’s head, filtering out the velocity of interest, and then extracting the periodic signal caused by heartbeats.

Both subtle color changes and head motions can be easily “hidden” by other signals during recording. The accuracy of HR estimation is influenced by the participants’ movements, complex facial features (face shape, hair, glasses, beards, etc.), facial expressions, camera noise and distortion, and changing light conditions. Many papers in this field use strictly controlled experiment settings to eliminate the influential factors. Besides well-controlled conditions, algorithms for noise reduction and signal recovery are applied to retrieve HR information. For intensity-based methods, averaging the pixel values inside a region of interest (ROI) is often applied to overcome sensor and quantization noise. Subsequently, temporal filters are adopted to extract the signal of interest (Poh et al., 2010; Wu et al., 2012). As for motion-based approaches, similar algorithms are used such as face tracking and noise reduction.

**Figure 2:**
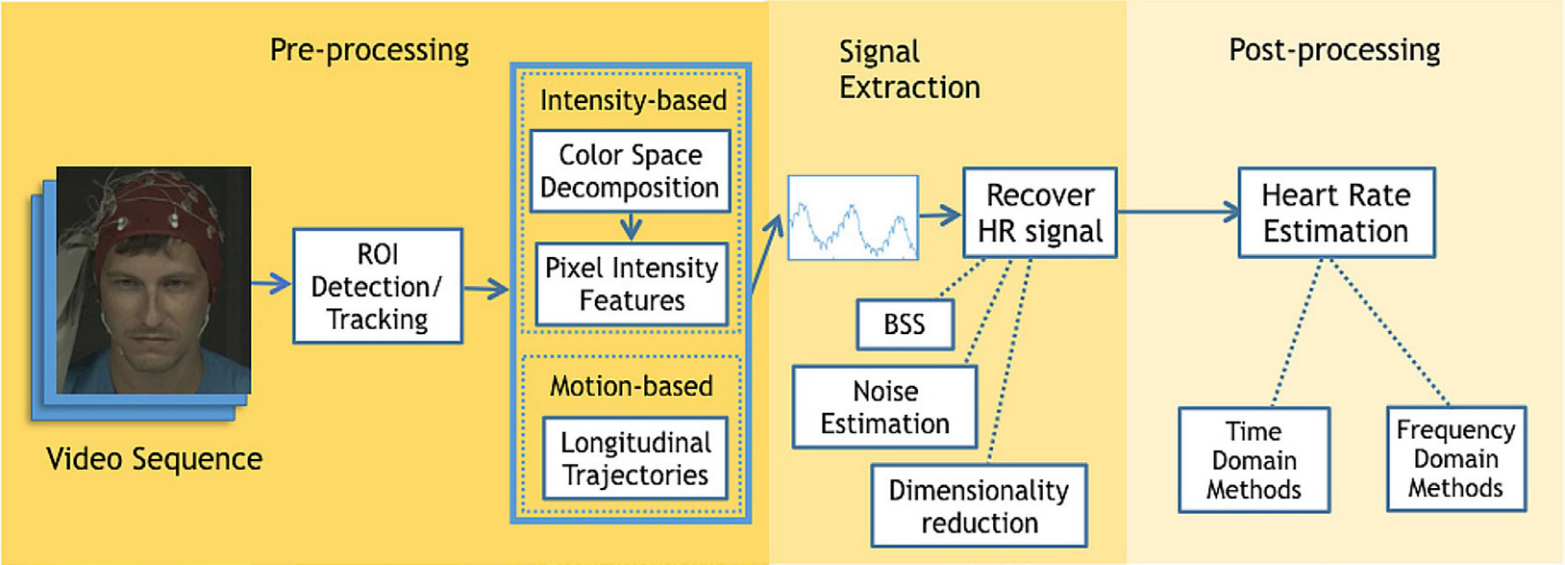
General schematic diagram for remote heart rate (HR) detection from face videos.

To categorize existing methods, we divide the HR detection procedure into three stages based on the implementation sequence: face video processing, face BVP signal extraction, and HR computation (Figure 2). Face video processing aims to detect faces, improve the motion robustness, reduce quantization errors, and prepare the featured signals for further BVP signal extraction. There are more algorithm variations at this stage than at BVP signal extraction and HR computation. For BVP signal extraction, temporal filtering, component analysis, and other approaches are used to recover HR information from noisy signals. The HR computation stage aims to compute HR from the cardiac signal obtained from the previous stage. At this stage, the methods can be grouped into time domain analysis and frequency domain analysis. For the time domain processing, peak detection is diffusely applied to get the inter-beat interval (IBI) from which HR is computed. In frequency domain, the power spectral density is mostly used, where the dominant frequency is taken as HR. HR computation can become complex for applications including buffer handling functions to present HR results after a certain time period (Stricker et al., 2014).

### Experiment Setting

**Figure 3:**
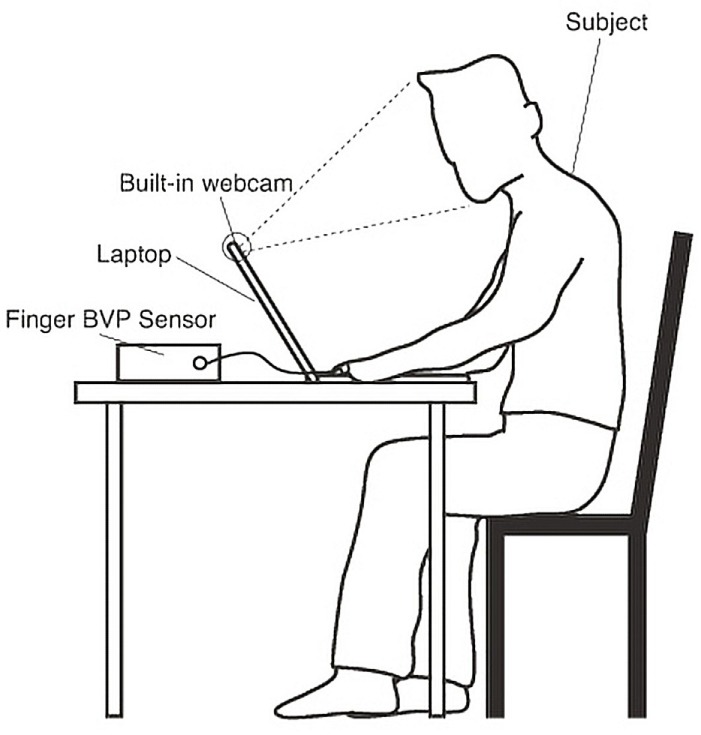
Experimental setup (Poh et al., 2010).

Only a few papers used public datasets for remote HR estimation from face videos (Li et al., 2014; Werner et al., 2014; Lam and Yoshinori, 2015; Tulyakov et al., 2016). While other researchers gathered their own datasets, where experiment settings vary substantially from camera settings, lighting situations to ground truth HR measurements as shown in Appendix I in Supplementary Material. The experimental setting often consists of placing a stable digital video camera in front of the participant under a controlled lighting condition. Furthermore, a ground truth HR measurement is also collected using a more traditional method. Figure 3 shows an example of the experimental setting. Finger BVP serves as the ground truth (13 out of 42 papers), while the face recordings are captured by the built-in camera of a laptop computer.

The digital cameras used for capturing videos are mainly commercial cameras like web cameras or portable device cameras. Following the Nyquist–Shannon sampling theorem, it is possible to capture HR signals at a frame rate of eight frames per second (fps), under the hypothesis that the human heartbeat frequency lies between 0.4 and 4 Hz. According to Sun et al. (2013), a frame rate between 15 and 30 fps is sufficient for HR detection. Among existing research, captured video frame rate differs from 15 (Poh et al., 2010) to 100 fps (Zaunseder et al., 2014). 30 fps, however, is the most often used within literature (Kwon et al., 2012; Pursche et al., 2012; Wei et al., 2012; etc.). It is important to note that for the majority of commercial digital cameras, the frame rate is not fixed. Sudden movements or illumination changes may force to drop or interpolate frames depending on the camera used. Frame rate is also closely related with frame resolution. Generally, cameras capture higher resolution frames at relatively low frame rates and *vice versa*. Both resolution and frame rate influence the HR estimation performance and computation load directly. By examining the table in Appendix I in Supplementary Material, it can be seen that the majority of research tends to utilize the video graphic array standard with a video resolution of 640 × 480 pixels per frame.

Illumination is strictly controlled for some experiments (De Haan and Vincent, 2013; Mestha et al., 2014; etc.) with specified fluorescent lights and no natural sunlight. There is also research using indirect sunlight only or fluorescent lights as supplementary. The distance between the tester and the camera highly depends on the lens properties. The distance is commonly set at 1.5 m to capture the entire face while minimizing the quantity of visualized background. In addition, the duration of recorded videos varies as well. Many face recordings are short-term with an approximate duration of 1 min. A setting description of references can be found in Appendix I in Supplementary Material.

### Face Video Processing

#### ROI Selection

Facial ROI selection is used to obtain blood circulation features and get the raw BVP signal, which highly influences the following HR detection steps. First, it affects the tracking directly since a commonly applied tracking method uses first frame ROI (Poh et al., 2010; Kwon et al., 2012; Pursche et al., 2012; etc.). Second, the selected ROI regions are regarded as the source of cardiac information. The pixel values inside a ROI are used for intensity-based methods, while feature point locations inside a ROI are used for motion-based methods. For intensity-based methods, when the selected region of the face is too large, the HR signal may be hidden in background noise. On the other hand, if the selected ROI is too small, the quantization noise caused by the camera may not be fully attenuated by the averaging of pixels intensity inside the ROI. For motion-based methods, significantly more computation time is required for a larger ROI. But there might not be enough feature points for effective motion tracking when the ROI is too small.

This step is similar for both intensity-based methods and motion-based methods (Figure 3). We classify the methods into two groups: box ROI detection and model-based ROI detection. Box ROI is the general area of the face regulated by a rectangle sometimes coupled with skin detection. While model-based ROI detection extracts the accurate face contours.

The easiest way of implementing a box ROI extraction is to manually select the desired area, such as the largest facial area with a rectangle as the bounding box on the first frame. This solution is applied for motion-based methods when the video resource contains solely hidden facial features, such as being covered by masks, or if the participants back is turned toward the camera (Balakrishnan et al., 2013). It is simple but highly subjective. Among automatic box face detection methods, the face detector proposed by Viola and Jones (2001) is often applied for HR detection (Poh et al., 2010; Balakrishnan et al., 2013; Irani et al., 2014; etc.). This method works rapidly and achieves reasonable detection accuracy which is 93.9% tested on MIT + CMU frontal face test set (Viola and Jones, 2001). To remove face edges and background area of the box ROI only part of the detected face area is used. According to Poh et al. (2010), 60% of width and full height of detected facial area are used. While Mestha et al. (2014) use the middle 50% of the rectangle’s width and 90% of its height. Some papers suggested to divide the roughly detected face region into a coarse grid with multiple ROIs, with the aim of removing the effect of head movements and facial expressions (Verkruysse et al., 2008; Sun et al., 2013; Kumar et al., 2015; Moreno et al., 2015). Skin detection methods are usually applied with other face detection solutions such as box ROI approach for HR estimation. Further details on this specific group can be consulted in the work of Vezhnevets et al. (2003), Xu et al. (2014), Sahindrakar et al. (2011), and Kakumanu et al. (2007).

Model-based approaches have been applied with accurate localization and tracking of facial landmarks (Werner et al., 2014; Lam and Yoshinori, 2015; Tulyakov et al., 2016). Datcu et al. (2013) uses a statistical method called active appearance model to handle shape and texture variation. In Stricker et al. (2014), deformable model fitting by regularized landmark mean-shift (Saragih et al., 2011) is applied. Li et al. (2014) applies a similar model named discriminative response map fitting with 66 facial landmarks inside the face region which is detected *a priori* by a Viola Jones face detector (Viola and Jones, 2001). Tulyakov et al. (2016) uses the facial landmark fitting tracker—Intraface (De la Torre et al., 2015). Alternatively to previously explored detection methods used in HR estimation, several other approaches exist such as OpenFace (Baltrušaitis et al., 2016), which can detect and track facial landmarks. Each area selected per frame is dynamic when using these model-based methods. Overall this makes the selection process more robust as it can vary in time based on the features themselves, increasing its efficiency when handling head motions and facial expressions. These algorithms, however, are more computationally expensive and time-consuming than box ROI detection. Further details on face detection methods can be found in Hjelmås and Low (2001), Vezhnevets et al. (2003), Rother et al. (2004), and Baltrušaitis et al. (2016).

#### Color Channel Decomposition

This step is specific for intensity-based methods. The basic idea is that the pixel intensity captured by a digital camera can be decomposed into the illumination intensity and reflectance of the skin. However, several approaches are proposed to relate the pixel value to the PPG signal. This can lead to various choices of color channel decomposition and combination. For example, Huelsbusch and Blazek (2002) separated the noise from the PPG signal by building a linear combination of two color channels to achieve motion robustness. An in-depth description of optical modeling can be found within the literature referenced in Table 1.

Color channels are based on the color models. There are mainly three color models applied in HR detection: Red-Green-Blue (RGB), Hue-Saturation-Intensity (HSI), and YCbCr where Y stands for luminance component and Cb and Cr refer to blue-difference and red-difference chroma components, respectively. The HSI model decouples the intensity component from the hue and saturation that carry color information of a color image. The skin-color lies in a certain range of H ([0 50]) and S ([0.23 0.68]) channels and the illumination changes information is separated in I channel. With each heartbeat, there is a clear drop in hue channel but its amplitude is very small. For HSI model, only the H channel can be used for BVP signal extraction. It is motion sensitive but performs better than RGB model without head motions. According to Sahindrakar et al. (2011), YCbCr produced better results in detecting HR than HSI with limited rotation and no transition. Among these three models, the most robust model is still RGB.

Among current detection methods, the main color space is still RGB, though some research criticizes that it intermixes the color and intensity information. According to Verkruysse et al. (2008), Stricker et al. (2014), and Ruben (2015), all channels contain PPG information, but the green channel gives the strongest signal-to-noise ratio (SNR). Consequently, the green channel has been the most popularly used for extracting HR (Verkruysse et al., 2008; Li et al., 2014; Zaunseder et al., 2014; Chen et al., 2015; etc.). However, Lewandowska et al. (2011) showed that the combination of the R and G channels contain the majority of cardiac information. Several research papers have also investigated the usage of all three color channels in conjunction with BSS for the BVP signal extraction (Poh et al., 2011; Kwon et al., 2012; Pursche et al., 2012; etc.).

#### Raw Featured Signal

Intensity-based methods use the intensity changes along the time as raw signal containing BVP information, while motion-based methods use the vertical component of the trajectories instead.

The spatial average is commonly employed in the majority of intensity-based methods, which aims to increase the SNR of PPG signals and enhance the subtle color changes (Verkruysse et al., 2008). Depending on the color channel selection, all pixels of the corresponding color channel within the ROI area are averaged at each frame. For a RGB video with *n* frames, the signal after spatial average can be expressed as a vector: *X*(*j*) = (*x*_1_(*j*), *x*_2_(*j*), …, *x_n_*(*j*)), *j* = 1, 2, 3 where *j* stands for the color channels. This method is simple and efficient to get raw featured signals for intensity-based methods. Several research papers used the spatial-averaged signal directly, as shown in Table 1. On the other hand, some works (De Haan and Vincent, 2013; Tulyakov et al., 2016) apply optical models and use the chrominance features for HR estimation, which takes light transmission and reflection on skin into consideration.

For motion-based methods, the location of time-series *x_k_*(*n*), *y_k_*(*n*) for each feature point *k* on frame *n* is tracked. Only the vertical component *y_k_*(*n*) is taken to extract the trajectory from each feature point. The longitudinal trajectories are then used as raw featured signals.

### Face BVP Signal Extraction

Now that the feature signal has been obtained from face videos, the heartbeat can be effectively extracted. This section is divided into two subsections exploring noise reduction and dimensionality reduction methods.

#### Noise Reduction

As previously explored, color and motion changes caused by the cardiac activities are often noisy. Thus this step is applied on the raw signals to remove such changes in light and tracking errors. For intensity-based methods, the light variations are recorded together with intensity changes caused by blood pulses (Verkruysse et al., 2008; Li et al., 2014; Zaunseder et al., 2014; etc.). For motion-based methods, trackers capture trajectories that are not solely caused by heartbeats, thus it is necessary to apply noise reduction as well (Balakrishnan et al., 2013; Irani et al., 2014). We present noise reduction methods based on two categories: temporal filtering and background noise estimation. Temporal filtering contains a series of filters that remove irrelevant information and keep trajectories and color frequencies that are of interest. Background noise estimation uses the background to estimate the noise caused by light changes.

For temporal filtering, various temporal filters are applied to exclude and amplify low-amplitude changes revealing hidden information (Poh et al., 2010; Wang, 2017). It contains detrending, moving-average, and bandpass filters, which are often applied to reduce irrelevant noise (Li et al., 2014). A detrending filter aims to reduce slow and non-stationary trends of signals (Li et al., 2014). After applying a detrending filter, the low frequencies of the raw signal are reduced drastically. This method is as effective as a high-pass, low-cutoff filter with substantially less latency. The moving-average filter removes random noise with temporal average of consecutive frames. It can efficiently smooth the trajectories and sudden color changes caused by light or motions. Additional methods such as a bandpass filter can also be used to remove irrelevant frequencies. Bandpass filters can be Butterworth or other FIR bandpass filters in the literature. It can be a Butterworth filter (Balakrishnan et al., 2013; Irani et al., 2014; Osman et al., 2015; etc.) or other FIR bandpass filters (Li et al., 2014) with cutoff frequency of normal HR. The cutoff frequency could be 0.7–4 (Villarroel et al., 2017), 0.25–2 (Wei et al., 2012), or other values. The parameter setting for these three types of filters differs from papers. For example, Ruben (2015) applies a fourth order bandpass zero-phase Butterworth filter, while Irani et al. (2014) employs an eighth order Butterworth filter to flat pass band maximally. More temporal filtering information applied for HR detection can be found in the work of Yu et al. (2014) and Tarvainen et al. (2002).

Background noise estimation methods target intensity changes and are only suitable under some situations. It is based on the assumptions that (a) both the ROI and background share the same light source and (b) the background is static and relatively monotone (Li et al., 2014). Under these assumptions, the intensity changes in the background are caused by illumination only and are correlated with the light noise in the HR signal extracted from face recordings. Adaptive filters are applied on noised HR signal and background signal to remove the noise (Chan and Zhang, 2002; Cennini et al., 2010; Li et al., 2014).

Once filtered, signals can be used either directly for post-processing or for further signal extraction (dimensionality reduction). If the signal is used directly, the green channel is mostly used since it contains the stronger PPG signal (Verkruysse et al., 2008). Under the second case, all the signal channels are kept.

#### Dimensionality Reduction Methods

The BVP signal is a periodic one-dimensional signal in the time domain. Dimensionality reduction algorithms are used to reduce the dimensionality from raw signals in order to more clearly reveal BVP information. The main idea is to find a mapping between higher dimensional space, such as three-dimensional RGB color spaces and one-dimensional space uncovering cardiac information. Dimensionality reduction contains classic linear algorithms, e.g., BSS methods [e.g., independent component analysis (ICA) and principle component analysis (PCA)], linear discriminant analysis, and manifold learning methods such as Isomap, Laplacian Eigenmap (LE), and locally linear embedding (Zhang and Zha, 2004). Wei et al. (2012) tested nine commonly used dimensionality reduction methods on RGB color channels and the result demonstrated that LE performs best for extracting BVP information on their dataset.

##### Blind Source Separation

After Poh’s publication in 2010, the mainstream technique to recover the BVP signal has been BSS which assumes that the observed signals (in our case the featured signals) are a mixture of source signals (BVP and noise). The goal of BSS is to recover the sources signals without or with a little prior information about their properties. The most popular BSS methods for HR detection from face video are ICA (Hyvärinen and Oja, 2000) and PCA (Wold et al., 1987; Abdi and Williams, 2010).

Independent component analysis is based on the assumption that all the sources are mutually independent. The basic principle is to maximize the statistical independence of all observed components in order to find the underlying components (Liao and Carin, 2002; Yang, 2006). For cardiac pulse detection, the observed signals are captured by camera color sensors, which are mixed with the heartbeat signals. Among various ICA algorithms, Joint Approximate Diagonalization of Eigen-matrices (JADE) (Cardoso, 1999) is popular for HR detection since it is numerically efficient in computation (Poh et al., 2010, 2011; Kwon et al., 2012; Pursche et al., 2012; etc.). JADE is a high-order measures of independence for ICA. Further details on the JADE algorithm can be found in the work of Hyvärinen and Oja (2000), while methods for optimizing JADE is further described by Kumar et al. (2015).

Principle component analysis can be used to extract both the intensity-based pulse signal and the head longitude trajectories caused by pulse (Lewandowska et al., 2011; Balakrishnan et al., 2013; Rubinstein, 2013). For motion-based method, the frequency spectra of the PCA components with the highest periodicity is selected quantified from spectral power, meanwhile the component with maximum variance is selected for intensity-based method as BVP signal (Lewandowska et al., 2011). Compared with ICA, PCA has lower computation complexity. PCA is concerned with finding the directions along which the data have maximum variance in addition to the relative importance of these directions. For HR detection, the goal of applying PCA is to extract the cardiac pulse information from the head motions or pixel intensity changes, represented it into principal components consisting of a new set of orthogonal variables (Abdi and Williams, 2010; Balakrishnan et al., 2013). Mathematically, PCA depends on the Eigen-decomposition of positive semi-definite matrices and the singular value decomposition of rectangular matrices (Wold et al., 1987).

### HR Computation

Once the BVP signal is effectively extracted, a post-processing procedure follows. HR can be estimated from time domain analysis (peak detection methods) or frequency domain analysis. Signals can be transformed to the frequency domain using standard methods, such as the Fast Fourier Transform (FFT) and discrete cosine transformation (DCT) methods. Currently, supervised learning methods are only applied at this stage for both the time and frequency domains.

Frequency domain algorithms are the most common post-processing methods within the literature. The extracted HR signal is converted to the frequency domain either by FFT (Poh et al., 2010; Pursche et al., 2012; Yu et al., 2013; etc.) or DCT (Irani et al., 2014). Using these methods, there is an assumption that the HR is the most periodic signal and thus, has the highest power of the spectrum within the frequency band corresponding to normal human HR. The drawback is that it can only compute the HR over a certain period instead of detecting instantaneous HR changes.

Peak detection methods (Poh et al., 2011; Li et al., 2014) detect the peak of HR signals in the time domain directly. With the detected peaks, IBI can be calculated. The IBI intervals are then averaged and the HR is computed from the average IBI. IBI allows for the beat-to-beat assessment of HR, however, it is quite sensitive to noise. To achieve more reliable results, a sliding window of short-time period is often implemented to average the HR result over the whole video (Li et al., 2014; Ruben, 2015).

Supervised learning methods have also been investigated as a potential solution for HR calculation (Monkaresi et al., 2014; Tarassenko et al., 2014; Osman et al., 2015). Monkaresi et al. (2014) and Tarassenko et al. (2014) both use supervised learning method for power spectrum analysis. With features extracted from PSD, auto-regression or *k*-nearest neighbor classifier is used to predict the HR signal with a degree of accuracy. Osman et al. (2015) extract the first order derivative of green channels as the features. A feature at time *t* is positively labeled, if there is a ground truth BVP peak lies within a certain time tolerance and *vice versa*. These data are then used to train a support vector machine algorithm, capable of predicting IBI.

### Discussion

Developed methods tend to be strongly tied into the dataset and the specific experiment protocol they were designed for. Unfortunately, this means that they neither generalize nor adapt to other datasets or scenarios, especially real-life situations. This section analyses the limitation aspect from setting to results.

#### Experiment Setting

As shown in Section “Experiment Setting,” experimental settings can vary significantly. For most experiments, both the participants’ behaviors and the environment are well-controlled which are not applicable for practical use. Non-grid motions like facial expressions are more difficult to handle compared with grid motions, e.g., head rotate horizontally or vertically. Detecting HR from face videos with spontaneous facial expressions is valuable for further study such as long-term monitoring and affective computing.

So far, there is no research designed specifically to test each influential factor of experimental setting such as video resolution, frame rate, and illumination changes. Consequently, it is difficult to distinguish whether the study results are affected by the experimental setting or the implemented approach. For example, low video resolution will lead to a limited number of pixels in the face area, which may be insufficient to extract the BVP signal. A high frame rate provides more information but may increase the computational load.

Furthermore, most of the self-collected datasets are not publicly accessible, complicating further investigations as researchers must continuously collect and construct new datasets, which can be time consuming. In fact, it complicates evaluation as methods are tested cross-dataset. A few studies used the open dataset MAHNOB-HCI (Li et al., 2014; Lam and Yoshinori, 2015; Tulyakov et al., 2016). However, their results for the same method are not consistent with each other (Li et al., 2014; Lam and Yoshinori, 2015). This is probably due to differences in the implementation.

Besides, participants with darker skin tone are also rarely used within both self-collected and open datasets. The higher amount of melanin present in darker skin tones absorbs a significant amount of incident light, and thus degrades the quality of the camera-based PPG signals, making the system ineffective for extracting vital signs (Kumar et al., 2015). Equipment limitations also exist. For example, the majority of digital cameras are unable to hold stable frame rates during recording, which can impact HR computation. Video compression is another factor that can influence performance (Ruben, 2015). According to McDuff et al. (2017), even with a low constant rate factor to compress videos, the signal-to-noise ratio degrades considerably in face BVP signals.

#### Method Discussion

The ROI definition is important since it contains the raw BVP signals. However, as mentioned in Section “ROI Selection,” there is still no consensus on which ROIs are the most relevant for HR computation. For example, Pursche et al. (2012) claim that the center of the face region provides better PPG information compared with other facial parts. By contrast, Lewandowska et al. (2011) and Stricker et al. (2014) assert that the forehead can represent the whole facial region although it can be unreliable if covered with hair. In Datcu et al. (2013), cheek and forehead are suggested as the most reliable parts containing the strongest PPG signals. Moreno et al. (2015) believe that forehead, cheeks, and mouth area provide more accurate heartbeat signals, in comparison with other parts such as nose and eyes. While Irani et al. (2014) state that the forehead and area around the nose are more reliable. These differences are caused specifically by the datasets used. For example, with videos containing head motions and facial expressions (Lewandowska et al., 2011; Stricker et al., 2014), the eyes and mouth areas tend to be less stable than the forehead area, since they are influenced by facial muscles.

The most popular method to extract the face BVP signal is by far ICA, as previously detailed in Section “Face BVP Signal Extraction.” It works experimentally, nevertheless, it also has some limitations. Based on the Beer–Lambert law, reflected light intensity through facial tissue varies nonlinearly with distance (Wei et al., 2012; Xu et al., 2014), while both PCA and ICA assume that the observed signal is a linear combination of several sources. Supporting the possibility that ICA is not the best option, Kwon et al. (2012) demonstrated that ICA performance is slightly lower compared with simple green channel detection methods. Besides, according to Mestha et al. (2014) and Sahindrakar et al. (2011), ICA needs at least 30 s to be accurately estimated and cannot handle large head motions. The noise estimation method proposed by Li et al. (2014) can only be applied with monotone backgrounds. Also, it is not suitable for real-time HR prediction with its adaptive filter, which needs a certain time duration to guarantee estimation accuracy. For stable frontal face videos, the green channel tends to be a good solution, and provides a low computational complexity method for the extraction of BVP signals. Particularly noisy sources of the face can often be mitigated through the usage of methods like PCA or ICA, and can often achieve better results than using the green channel exclusively (Poh et al., 2011; Li et al., 2014).

At the HR computation stage, the frequency domain methods are not capable to detect instantaneous heartbeat changes, and are not as robust as time domain methods according to Poh et al. (2011). Supervised learning methods are mainly (three out of four papers) applied at this stage so far. There is one recent paper apply auto-regression to extract BVP signals after the video processing (Villarroel et al., 2017), but there is no end-to-end usage of supervised learning methods. Future research might thus focus on the development of machine learning methods trained to take raw videos as inputs and compute the HR information as outputs. Without inter-processing stage, the performance of supervised learning methods may be improved significantly.

#### HR Estimation

The HR discussed in this article is actually the average of HR during a certain time interval (e.g., 30 s or 1 min), which cannot reveal the instantaneous physiological information. The literature often computes HR by examining videos with various lengths and subsequently calculating the average HR over that time period. In reality, the time interval between two connective heartbeats is not stable. By contrast to averaged HR, we refer to instantaneous or dynamic HR when talking about HR calculated for each IBI. This information can be used to reveal short lived phenomenon such as emotions. It can further be used to compute heart rate variability (HRV). According to Pavlidis et al. (2007) and Dawson et al. (2010), HRV is directly related with emotion and disease diagnosis, which is of great value in medical and affective computing domain.

To the best of our knowledge, so far there is no work focusing on dynamic HR. Also, for average HR estimation, there are no state-of-the-art approaches that are robust enough to be fully operated under real situations with grid and non-grid movements, illumination changes and noise caused by the camera. Even with well-established public dataset, one or two influential factors are still under controlled.

#### Commercial Applications and Software

Currently, for application purposes, PPG is mainly obtained from contact sensors. There are few commercial applications and software available estimating HR remotely from the color changes on faces such as Cardiio,^1^ Pulse Meter,^2^ and Vital Signs Camera.^3^ Cardiio and Pulse Meter are both phone applications. They present a circle or a rectangle on the screen for users to place their face areas and keep still for a certain time period. Vital sign camera is developed by Philips with both software and phone application. They apply frequency domain method to calculate HR. All these applications and software compute the average HR only and require users to stay stable. None of these products provide an estimation of their performance.

## Comparative Analysis

It is of great importance to quantify and compare the performance of the main algorithms presented in the previous section. In this section, we study how the existing approaches perform in a close to realistic scenario with both gird and non-grid head movements.

Given the amount of workload, not all methods were tested exhaustively. For motion-based methods, only two methods were proposed and applied on face videos (Balakrishnan et al., 2013; Irani et al., 2014). Both of them required limited head movements. Our test dataset, MANNOB–HCI, is not ideal for this group of methods. Therefore, we focus on the validation of intensity-based approaches which are most often applied. Despite of this restriction, some methods evaluated in this section can also be used as reference for motion-based methods. This is for instance the case for ROI selection which can be used in a motion-based framework.

To offer a panoramic view of the state-of-the-art, this article attempted to cover the analysis with methods from different categories within each processing stage as previously stated in Section “Remote Methods for HR Detection.” The methods considered for comparative analysis are selected based on their popularity (times that they were adopted for other papers) and their category (supervised learning, dimensionality reduction, etc.).

**Figure 4:**
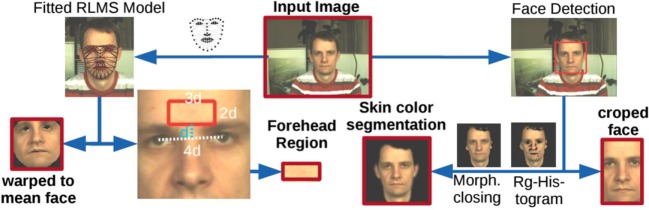
Overview of region of interest selection (Stricker et al., 2014).

The method validation is divided into two parts. In the first part, we test and compare the algorithms at each stage sequentially as shown in Figure 4. For one stage, we test several alternatives while keeping the algorithms applied at the other two stages fixed. Once a stage has been tested, the best method on this stage will be chosen for the following stage test. Before that, the algorithms selected for fixed stages are based on simplicity. At the pre-processing stage, the main target is to find out the efficient face segmentation. For signal extraction, various algorithms are tested to compare the most efficient method capable of separating the HR signal from noise and irrelevant information. At the post-processing stage, time domain and frequency domain methods are compared for HR computation. The second part focuses on the implementation of state-of-the-art methods presented in the work of Poh et al. (2011), Li et al. (2014), and Osman et al. (2015), which are used as baselines for validation.

The state-of-art methods were tested on the MAHNOB-HCI (Soleymani et al., 2012) database. In MAHNOB-HCI, 27 participants (15 females and 12 males) were recorded while watching movie clips to elicit emotions, which can influence their HR and stimulate facial expressions. This public dataset is multimodal including frontal face videos and HR information recorded using a gold standard technique: ECG. The frame rate is 61 fps and the ECG sampling frequency is 256 Hz. Furthermore, this dataset is publicly accessible, allowing research to be easily reproduced. We filtered 465 samples (i.e., ECG and facial videos which each correspond to an emotion-elicited movie clip) from MAHNOB-HCI obtained from 24 participants, where 12 were males and 12 were females.

To reduce the possible risks of bias, we regulated the study from data source to performance validation. The face videos used for the study are from both genders and different skin tones. For one participant, 14–20 videos are used to avoid bias from a certain scenario due to a specific stimulus. All participants we selected offered their consent before experimentation, where the recording duration surpasses 65 s. Among the 465 samples, 20 samples did not contain corresponding ECG signals and 1 recording presented faulty filtering. These samples were removed leaving a total of 444 samples. The validation followed by Li et al. (2014) and Lam and Yoshinori (2015) started each video recording with a delay of 5 s. Videos are subsequently cut into 30 s segments, and subsequently synchronized with their corresponding ECG signal. All the methods and evaluations are implemented using MATLAB R2016a. To validate obtained results, the HR ground truth is obtained by first detecting the R peaks from ECG signals using the TEAP (Soleymani et al., 2017), which uses the standard Tompkins’ method (Tompkins, 1993). Absent and falsely detected peaks are then manually corrected. The mean HR is the averaged value computed from the instantaneous HR. Thus, a precise average HR is guaranteed as ground truth.

### Comparison at Each Stage

#### Face Segmentation

**Figure 5:**
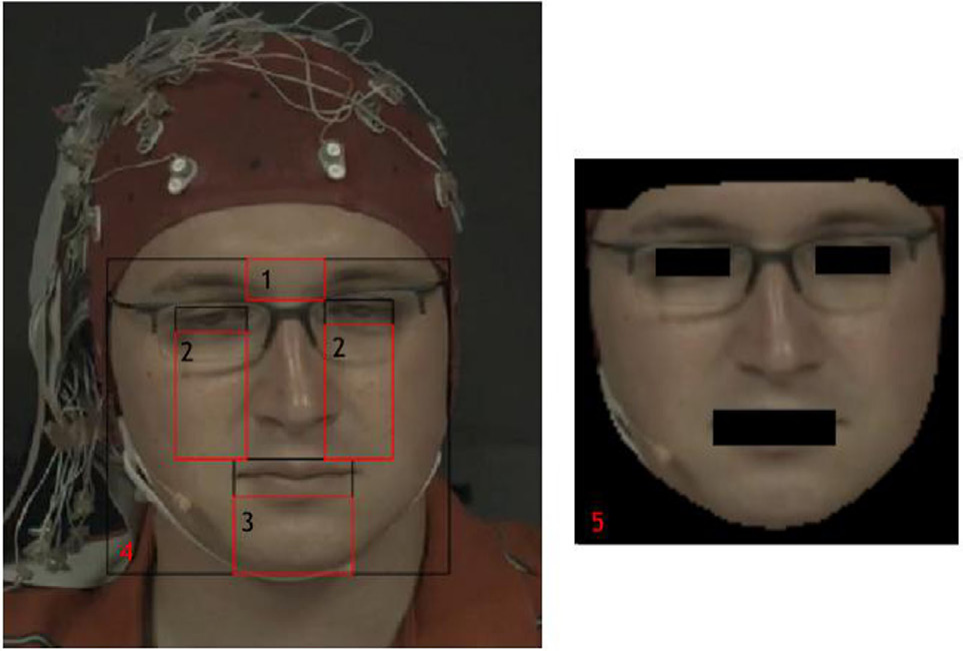
Face segmentation (1. Forehead; 2. Cheeks; 3. Chin; 4. Whole face; 5. Extracted skin).

As shown in Section “Face Video Processing,” there is no consistent conclusion about which part of the face reveals most HR information. Thus, there is an interest in comparing HR detection accuracy for several facial regions. The face region contains useful features of HR information that may differ from every frame since appearance changes are spatially and temporally localized. Instead of using a constant and preselected ROI, we adopt OpenFace (Baltrušaitis et al., 2016) to detect face segmentations automatically and dynamically. OpenFace can extract 66 landmarks from the face, marking the location of eyes, nose, eyebrows, mouth, and the contour of visage. We compared the performance of forehead, cheeks, chin, whole face, and extracted skin area (accurate face contour area without eyes and mouth), which are frequently selected as ROIs in the state-of-the-art. For the rectangular forehead area, we used the distance between inner corners of the eyes as the rectangle width, while the distance from the uppermost face contour landmark to the uppermost eyes landmark constitutes the rectangle height. Similarly, the cheek areas had the same width as eyes and the height is the distance between upper lip border and the lower eye border. For the skin area, we removed the eye and mouth regions to avoid noise caused by blinks and other facial expressions. The regions we tested are shown in Figure 5. Since all the five ROI selections are determined by facial landmarks, the ROI areas are dynamic and may change due to the head motions from frame-to-frame. Given that in the MAHNOB-HCI database, the participants’ electroencephalogram (EEG) was recorded using a head cap, the forehead area was partially covered by the sensors that may influence the performance.

To avoid influential factors from other processing steps, we use the spatial-averaged green channel directly of each ROI and then apply temporal filtering to obtain the HR signal. The cutoff frequency is set from 0.7 to 2 Hz which corresponds to HR between 42 and 120 bpm. The PSD method (Poh et al., 2010) is applied to calculate the averaged HR over 30 s.

#### Face BVP Signal Extraction

For this part, we tested the main methods mentioned in Section “Noise Reduction.” Temporal filtering with a detrending filter and a bandpass filter are applied on the raw featured signal before methods testing in this section. Three signal extraction methods were compared. PCA, ICA, and background noise estimation methods are evaluated on spatial-averaged RGB signals from extracted skin ROI. For ICA, the component with highest energy in the frequency domain is selected as the BVP signal, while the component with the highest variance is selected for PCA. The implementation of ICA and PCA follows Poh et al. (2011) and Rubinstein (2013), respectively. Background noise estimation is implemented following Li et al. (2014) and uses green channel for extracting HR signals after illumination rectification with normalized least mean square adaptive filter. HR is estimated using the PSD of the extracted face BVP signal (Poh et al., 2010) as well.

#### HR Computing

The peak detection method and PSD method are evaluated for the calculation of HR. Selected ICA components extracted from skin ROI are used as the HR signal. For peak detection, we applied the algorithm from the open source Toolbox for Emotional feAture extraction from Physiological signals (TEAP) (Soleymani et al., 2017). There are different ways of computing PSD. In this section, it is estimated *via* the periodogram method following Monkaresi et al. (2014).

### Comparison on Complete Methods

**Figure 6:**
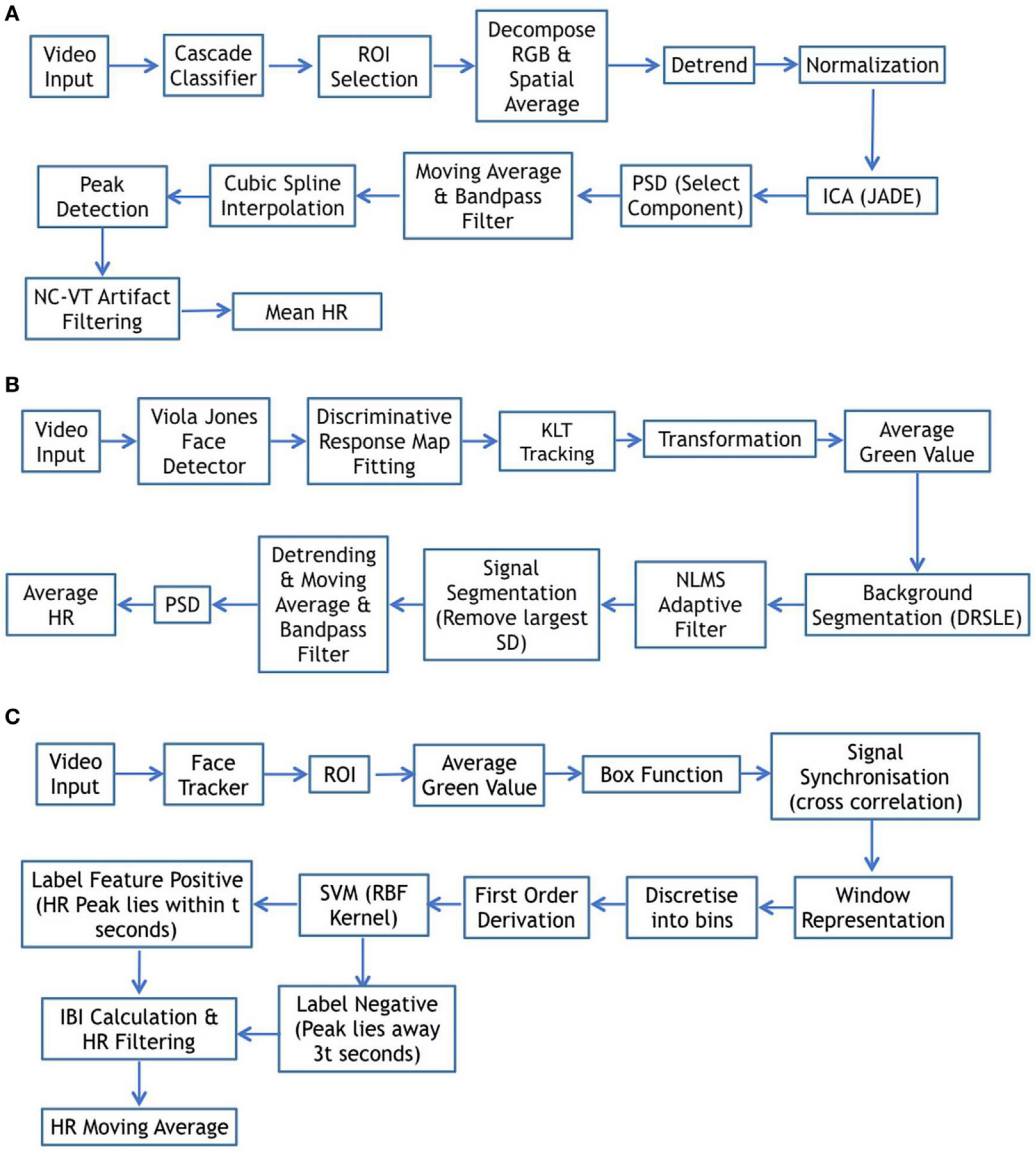
Schematic diagram for complete method comparison. **(A)** Schematic diagram from Poh et al. (2011). **(B)** Schematic diagram from Li et al. (2014). **(C)** Schematic diagram from Osman et al. (2015).

We reproduced three methods from the work of Poh et al. (2011), Li et al. (2014), and Osman et al. (2015). This was done to compare our analysis with the three main categories of HR estimation methods: ICA, background noise estimation, and machine learning, respectively. We implement these three approaches step-by-step and set parameters as mentioned in the papers. The implementation schematic is shown in Figure 6.

Poh et al. and Osman et al. tested their method on self-collected datasets with BVP signals as ground truth, while Li et al. used both self-collected and the MAHNOB-HCI datasets.

The work of Osman et al. (2015) used finger BVP signals to label their extracted features. In our reproduction, ECG signals are used from MAHNOB-HCI. We followed the same method but labeled the feature positive if the R peak existed within the time tolerance of the detected peak from face videos. We randomly selected 12 subjects as training dataset and the other 12 subjects as testing dataset. For training, we have 5,000 positive features and 5,000 negative features as Osman et al. (2015). For testing, there are 6,765 positive features and 7,127 negative ones.

## Results and Discussion

### Results at Each Stage

**Figure 7:**
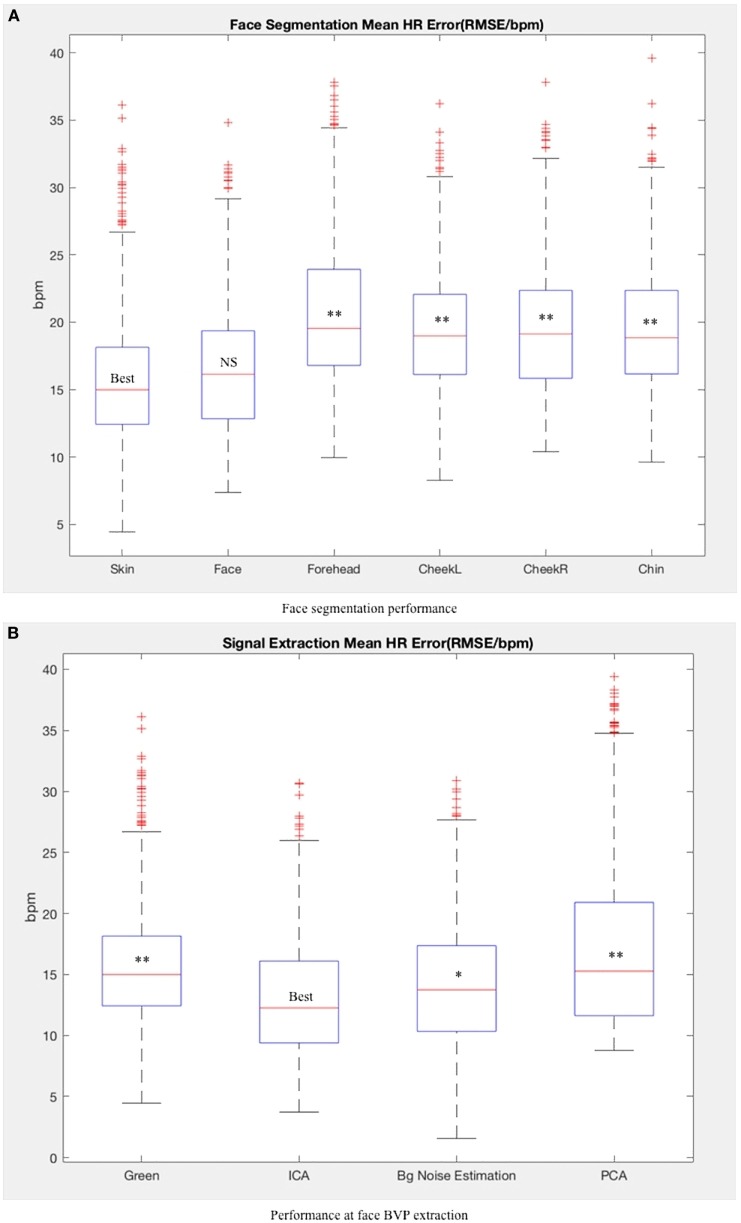

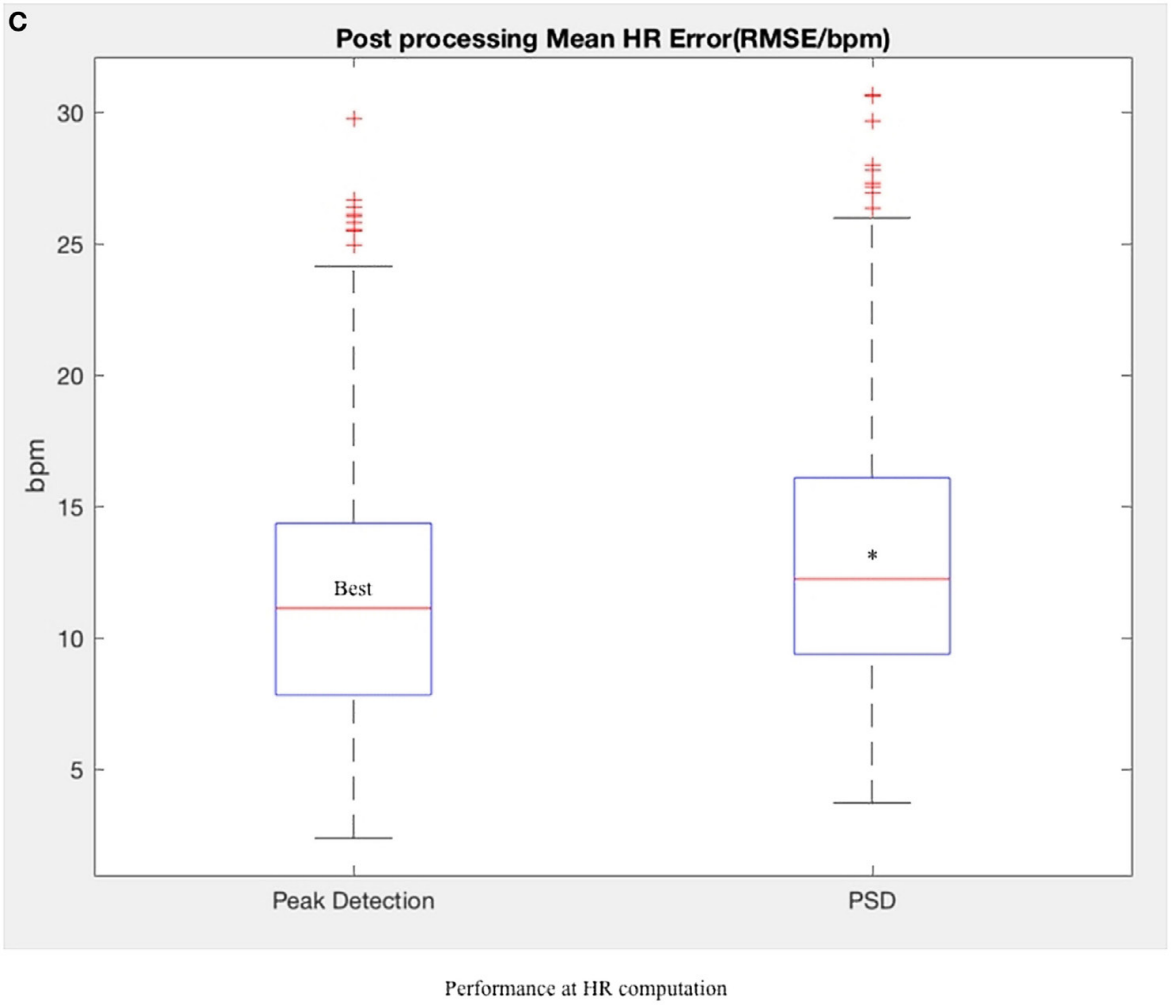
Method performance at each stage. “best” indicate the best methods according to average performance. Other methods are tested against the best method (ns, non-significant; *, *p* < 0.05; **, *p* < 0.01). **(A)** Face segmentation performance. **(B)** Performance at face blood volume pulse (BVP) extraction. **(C)** Performance at heart rate (HR) computation.

For face segmentation, we can see from Figure 7A that the extracted skin area performs better than the other facial areas, followed by the entire facial region. Though skin area performs a bit better than face area, there is no significant difference between them (*t* = 1.72; *p* = 0.07). There is no noticeable difference between the left cheek and the right cheek and the forehead does not perform better than other facial areas. According to our results, the more skin area used as BVP resource, the better performance we can achieve (extracted skin area performs better than cheek, forehead, and chin areas). The facial expressions, such as laugh and blinks, tends to add extra noise to the BVP signals but does not influence it considerably when taking the whole face into consideration. We also compared the whole face area with the area detected by Viola Jones face detector (Viola and Jones, 2001). When there are no abrupt head moments and other interruptions, the featured raw signals obtained from these two methods are very similar. The Pearson correlation is statistically significant (*r* = 0.99; *p* < 0.001). When the video includes more spontaneous movements, OpenFace is more robust with a higher success detection rate and the correlation between the two methods is degraded (*r* = 0.83; *p* < 0.001). Viola Jones detector fails face detection on several frames and then uses the last successfully detected face information which adds noise in face BVP signal. Considering the computation complexity and detection efficiency, Viola Jones face detector is suitable for slight head movements and can be used as a prior method for more complex face detection algorithms.

To see how head movements influence signal extraction, we tested the methods on one video with little head motions. For this situation, background noise estimation performs better since the illumination was the main source of noise. However, there is little difference between the root mean squared error of the green channel (12.12), PCA (12.51), ICA (11.80), and background noise estimation (11.69). Evaluating on all the samples, ICA performs better then background noise estimation (*t* = 2.27; *p* = 0.04) and PCA (*t* = 4.84; *p* < 0.001) as shown in Figure 7B. Under the experimental setting of MAHNOB-HCI, head motions tend to have a higher influence on the HR detection, rather than illumination changes.

For HR computation, Figure 7C shows that the implemented peak detection method is more robust than the PSD method (*t* = 2.52; *p* = 0.03). Peak detection reduces the error by averaging all the IBI over the video duration. For PSD, once the noise takes the dominant frequency and lies in the human HR range, there is no solution to detect the right HR. The best result from each stage is shown in Table 2 with extracted skin area, ICA and peak detection method.

**Table 2:** Obtained performance for the best method at each stage.

Stage	M (SD)/bpm	RMSE/bpm	ρ
Face video processing (extracted skin area)	5.34 (14.98)	15.05	0.20
Blood volume pulse signal extraction (independent component analysis)	4.09 (13.37)	13.56	0.32
Heart rate computing (peak detection)	3.01 (12.14)	12.23	0.55

*Performance is measured by M (mean error), SD (standard derivation), RMSE (root mean squared error), and ρ (correlation coefficient)*.

### Results From Complete Methods

The results of three methods tested on MAHNOB-HCI are shown in Table 3. Unsurprisingly the performance of Poh et al. (2011)’s method is significantly dropped than its self-reported results using the proprietary dataset (mean bias is 0.64, RMSE is 4.63, and correlation coefficient is 0.95). This is probably due to the fact that the original dataset is rather stationary and avoids head motions. Li et al. (2014) and Lam and Yoshinori (2015) both tested the Poh et al. (2011) method on MAHNOB-HCI dataset. Our testing result is a bit worse than Li et al. (2014) whose mean bias is 2.04, RMSE is 13.6, and correlation coefficient is 0.36, but better than the result from Lam and Yoshinori (2015) (RMSE is 21.3). Following Li et al. (2014) method, we obtained better performance than Lam and Yoshinori (2015), but worse results than that presented by Li et al. (2014) themselves. Although evaluations are all based on the MAHNOB-HCI dataset, samples, and algorithm parameters are not exactly the same. As to the method from Osman et al. (2015), we cannot really compare the results since it was tested on self-collected dataset only. It shows better result than Poh et al. (2011), but not as good as Li’s method. From our test, the method of Li et al. (2014) performs significantly better than Poh et al. (2011) (*t* = 5.00; *p* < 0.001) and Osman et al. (2015) (*t* = 4.51; *p* < 0.001).

**Table 3:** Obtained performance for the complete methods.

Method	M (SD)/bpm	RMSE/bpm	ρ
Poh et al. (2011)	4.07 (13.04)	13.81	0.28
Li et al. (2014)	2.15 (10.04)	10.33	0.68
Osman et al. (2015)	3.37 (12.08)	12.79	0.47

*Performance is measured by M (mean error), SD (standard derivation), RMSE (root mean squared error), and ρ (correlation coefficient)*.

We can see from Tables 2 and 3 that the face segmentation influences results significantly. With the best performed method of each stage, it can achieve competitive results compared with complete state-of-the-art methods which are more complex. Thus, we obtained and proposed an efficient pipeline with extracted skin area, ICA and peak detection for detecting HR remotely from face videos.

This pipeline could be applied for other studies with front face recordings under environmental illumination. It is robust with facial expressions and limited head motions (translation and orientation), which is often the case for the majority of human–computer interaction processes (online education, computer gaming, etc.). The pipeline is expected to perform better under more stable conditions with less head movements. Interruptions such as hands partially covering the face will influence the performance of this method. Furthermore, if the illumination changes significantly during the HR detection process, noise estimation methods could be applied to improve the overall performance.

The main limitation of this study comes specifically from the selected database and testing methods. Possible risks of bias can derive from the small number of subjects (24 participants) and the selected data source (444 videos and corresponding ECG signals from MAHNOB dataset). The specific experimental setting could favor some of the considered methods and could potentially be detrimental for others. For example, the forehead area is reported have a good performance (Lewandowska et al., 2011; Stricker et al., 2014), while our results showed that this was not significant, potentially due to the EEG head cap which partially covers the forehead area in MAHNOB videos.

## Conclusion

Remote HR measurements from face videos have improved during the last few years. Among the research in this domain, the designed models, parameter settings, chosen algorithms, and equipment are plenty, complex, and vary enormously. Some approaches achieve high accuracy under well-controlled situations but degrade with illumination changes and head motions. In this article, we performed (a) the collection and classification of state-of-the-art methods into three stages and (b) the comparison of their performance under HCI conditions.

The MAHNOB-HCI dataset is used for algorithm testing and analysis since it is a publicly accessible dataset. Our results showed that at the pre-processing stage, accurate face detection algorithms performed better than rough ROI detection. The extracted facial skin area used as the source HR signal obtained better result than any other facial ROI. For signal extraction, under most cases, ICA method obtained decent results. When the background is monotone, removing the noise estimated from the background increased the HR detection accuracy efficiently. As for post-processing, peak detection in time domain was more reliable than the frequency domain methods. In conclusion, we built an efficient pipeline for non-intrusive HR detection from face videos by combining the methods we found to be the best. This pipeline with skin area extraction, ICA and peak detection demonstrated a state-of-the-art accuracy.

Though considerable progress has been made in this domain, there are still many difficulties. The state-of-the-art approaches are not robust enough when applied under natural conditions and are still unable to detect HR in real-time. Technically speaking, machine learning methods may be promising for remote HR detection. Especially with finger oximeter as ground truth measurement, since the BVP signal detected from facial regions should have a similar shape with collected finger or ear BVP signals. So far there are only three papers using machine learning methods and both of them concentrate on the post-processing category. With the development of deep end-to-end learning, the robustness and accuracy of HR detection may increase efficiently even under naturalistic situations.

Instantaneous HR reveals more information about the subject’s physiological and affective status than mean HR. According to Malik (1996) and Armony and Vuilleumier (2013), the heart beat variability can be used for investigating mental workload or to detect emotions such as anger and sadness. The work from Lakens (2013) has already shown the possibility of using smartphones to measure HR variation associated with relived experiences of anger and happiness. Thus psychological insights of HR changes and the link to affective status can be taken into consideration for further study. In short, more efforts could be devoted to reliable instantaneous HR detection in realistic scenarios.

## Author Contributions

CW designed and implemented the methods. CW and GC analyzed and interpreted the results. All authors contributed to the redaction of the manuscript.

## Conflict of Interest Statement

The authors declare that the research was conducted in the absence of any commercial or financial relationships that could be construed as a potential conflict of interest.

## Footnotes

^1^Cardiio. Cardiio. https://www.cardiio.com/ (Accessed: April 09, 2018).

^2^Rapsodo. Pulse Meter. http://www.appspy.com/app/667129/pulse-meter (Accessed: April 09, 2018).

^3^Philips. Philips vitals signs camera. http://www.vitalsignscamera.com (Accessed: April 09, 2018).

## References

[B1] Abdi H. Williams L. J. (2010). Principal component analysis. Wiley Interdiscip. Rev. 2, 433–459. 10.1002/wics.101

[B2] Allen J. (2007). Photoplethysmography and its application in clinical physiological measurement. Physiol. Meas. 28, R1. 10.1088/0967-3334/28/3/R01 17322588

[B3] Armony J. Vuilleumier P. (eds) (2013). The Cambridge Handbook of Human Affective Neuroscience. Cambridge University Press.

[B4] Balakrishnan G. Durand F. Guttag J. (2013). “Detecting pulse from head motions in video,” in Proceedings of the IEEE Conference on Computer Vision and Pattern Recognition Portland, Oregon.

[B5] Baltrušaitis T. Robinson P. Morency L.-P. (2016). “Openface: an open source facial behavior analysis toolkit,” in Applications of Computer Vision (WACV), 2016 IEEE Winter Conference on (IEEE).

[B6] Cardoso J.-F. (1999). High-order contrasts for independent component analysis. Neural Computat. 11, 157–192. 10.1162/089976699300016863 9950728

[B7] Cennini G. Arguel J. Akşit K. van Leest A. (2010). Heart rate monitoring via remote photoplethysmography with motion artifacts reduction. Opt. Exp. 18, 4867–4875. 10.1364/OE.18.004867 20389499

[B8] Chan K. W. Zhang Y. T. (2002). Adaptive reduction of motion artifact from photoplethysmographic recordings using a variable step-size LMS filter. Sensors 2, 1343–1346. 10.1109/ICSENS.2002.1037314

[B9] Chen D.-Y. Wang J. J. Lin K. Y. Chang H. H. Wu H. K. Chen Y. S. (2015). Image sensor-based heart rate evaluation from face reflectance using hilbert–huang transform. IEEE Sens. J. 15, 618–627. 10.1109/JSEN.2014.2347397

[B10] Chen W. Picard R. W. (2017). “Eliminating physiological information from facial videos,” in Automatic Face & Gesture Recognition (FG 2017), 2017 12th IEEE International Conference on (IEEE).

[B11] Da H. Winokur E. S. Sodini C. G. (2011). “A continuous, wearable, and wireless heart monitor using head ballistocardiogram (BCG) and head electrocardiogram (ECG),” in 2011 Annual International Conference of the IEEE Engineering in Medicine and Biology Society (Boston, Massachusetts: IEEE). 10.1109/IEMBS.2011.6091171 22255394

[B12] Datcu D. Cidota M. Lukosch S. Rothkrantz L. (2013). “Noncontact automatic heart rate analysis in visible spectrum by specific face regions,” in Proceedings of the 14th International Conference on Computer Systems and Technologies (New York: ACM).

[B13] Dawson J. A. Kamlin C. O. F. Wong C. Te Pas A. B. Vento M. Cole T. J. (2010). Changes in heart rate in the first minutes after birth. Arch. Dis. Childhood Fetal Neonatal Ed. 95, F177–F181. 10.1136/adc.2009.169102 20444810

[B14] De Haan G. Vincent J. (2013). Robust pulse rate from chrominance-based rPPG. IEEE Transac. Biomed. Eng. 60, 2878–2886. 10.1109/TBME.2013.2266196 23744659

[B15] De la Torre F. Chu W.-S. Xiong X. Vicente F. Ding X. Jeffrey Cohn J. (2015). “IntraFace,” in Automatic Face and Gesture Recognition (FG), 2015 11th IEEE International Conference and Workshops on, Vol. 1 (Pittsburgh: IEEE). 10.1109/FG.2015.7163082 PMC4918819 27346987

[B16] Garbey M. Sun N. Merla A. Pavlidis I. (2007). Contact-free measurement of cardiac pulse based on the analysis of thermal imagery. IEEE Trans. Biomed. Eng. 54, 1418–1426. 10.1109/TBME.2007.891930 17694862

[B17] Hjelmås E. Low B. K. (2001). Face detection: a survey. Comput. Vis. Image Understand. 83, 236–274. 10.1006/cviu.2001.0921

[B18] Huelsbusch M. Blazek V. (2002). “Contactless mapping of rhythmical phenomena in tissue perfusion using PPGI,” in Proc. SPIE 4683, Medical Imaging 2002: Physiology and Function from Multidimensional Images, Vol. 110, San Diego, CA.

[B19] Hyvärinen A. Oja E. (2000). Independent component analysis: algorithms and applications. Neural Networks 13, 411–430. 10.1016/S0893-6080(00)00026-5 10946390

[B20] Irani R. Nasrollahi K. Moeslund T. B. (2014). “Improved pulse detection from head motions using DCT,” in Computer Vision Theory and Applications (VISAPP), 2014 International Conference on, Vol. 3 (Lisbon, Portugal: IEEE).

[B21] Jeanne V. Asselman M. den Brinker B. Bulut M. (2013). “Camera-based heart rate monitoring in highly dynamic light conditions,” in 2013 International Conference on Connected Vehicles and Expo (ICCVE) (Las Vegas, NV: IEEE).

[B22] Jensen J. N. Hannemose M. (2014). Camera-based Heart Rate Monitoring. Lyngby, Denmark: Department of Applied Mathematics and Computer Science, DTU Computer, 17.

[B23] Kakumanu P. Makrogiannis S. Bourbakis N. (2007). A survey of skin-color modeling and detection methods. Pattern Recognit. 40, 1106–1122. 10.1016/j.patcog.2006.06.010

[B24] Kumar M. Veeraraghavan A. Sabharwal A. (2015). DistancePPG: robust non-contact vital signs monitoring using a camera. Biomed. Opt. Exp. 6, 1565–1588. 10.1364/BOE.6.001565 26137365 PMC4467696

[B25] Kwon S. Kim H. Suk Park K. (2012). “Validation of heart rate extraction using video imaging on a built-in camera system of a smartphone,” in 2012 Annual International Conference of the IEEE Engineering in Medicine and Biology Society (San Diego, CA: IEEE). 10.1109/EMBC.2012.6346392 23366353

[B26] Lakens D. (2013). Using a smartphone to measure heart rate changes during relived happiness and anger. Trans. Affect. Comput. 4, 238–241. 10.1109/T-AFFC.2013.3

[B27] Lam A. Yoshinori K. (2015). “Robust heart rate measurement from video using select random patches,” in Proceedings of the IEEE International Conference on Computer Vision Santiago, Chile.

[B28] Lewandowska M. Rumiński J. Kocejko T. Nowak J. (2011). “Measuring pulse rate with a webcam—a non-contact method for evaluating cardiac activity,” in Computer Science and Information Systems (FedCSIS), 2011 Federated Conference on (Szczecin, Poland: IEEE).

[B29] Li X. Chen J. Zhao G. Pietikainen M. (2014). “Remote heart rate measurement from face videos under realistic situations,” in Proceedings of the IEEE Conference on Computer Vision and Pattern Recognition Columbus, Ohio.

[B30] Liao X. Carin L. (2002). “A new algorithm for independent component analysis with or without constraints,” in Sensor Array and Multichannel Signal Processing Workshop Proceedings, 2002 (Rosslyn, VA: IEEE).

[B31] Malik M. (1996). Heart rate variability. Ann. Noninvasive Electrocardiol. 1, 151–181. 10.1111/j.1542-474X.1996.tb00275.x PMC6931824 29938866

[B32] McDuff D. J. Blackford E. B. Estepp J. R. (2017). “The impact of video compression on remote cardiac pulse measurement using imaging photoplethysmography,” in Automatic Face & Gesture Recognition (FG 2017), 2017 12th IEEE International Conference on (IEEE).

[B33] Mestha L. K. Kyal S. Xu B. Lewis L. E. Kumar V. (2014). “Towards continuous monitoring of pulse rate in neonatal intensive care unit with a webcam,” in 2014 36th Annual International Conference of the IEEE Engineering in Medicine and Biology Society (Chicago, IL: IEEE). 10.1109/EMBC.2014.6944455 25570823

[B34] Monkaresi H. Calvo R. A. Yan H. (2014). A machine learning approach to improve contactless heart rate monitoring using a webcam. IEEE J Biomed. Health Inform. 18, 1153–1160. 10.1109/JBHI.2013.2291900 25014930

[B35] Moreno J. Ramos-Castro J. Movellan J. Parrado E. Rodas G. Capdevila L. (2015). Facial video-based photoplethysmography to detect HRV at rest. Int. J. Sports Med. 36, 474–480. 10.1055/s-0034-1398530 25700104

[B36] Muender T. Miller M. K. Birk M. V. Mandryk R. L. (2016). “Extracting heart rate from videos of online participants,” in Proceedings of the SIGCHI Conference on Human Factors in Computing Systems (CHI’2016) San Jose, CA.

[B37] Osman A. Turcot J. El Kaliouby R. (2015). “Supervised learning approach to remote heart rate estimation from facial videos,” in Automatic Face and Gesture Recognition (FG), 2015 11th IEEE International Conference and Workshops on, Vol. 1 (Washington: IEEE).

[B38] Pavlidis I. Dowdall J. Sun N. Puri C. Fei J. Garbey M. (2007). Interacting with human physiology. Comput. Vis. Img. Understand. 108, 150–170. 10.1016/j.cviu.2006.11.018

[B39] Poh M.-Z. McDuff D. J. Picard R. W. (2010). Non-contact, automated cardiac pulse measurements using video imaging and blind source separation. Opt. Exp. 18, 10762–10774. 10.1364/OE.18.010762 20588929

[B40] Poh M.-Z. McDuff D. J. Picard R. W. (2011). Advancements in noncontact, multiparameter physiological measurements using a webcam. IEEE Trans. Biomed. Eng. 58, 7–11. 10.1109/TBME.2010.2086456 20952328

[B41] Pursche T. Krajewski J. Moeller R. (2012). “Video-based heart rate measurement from human faces,” in 2012 IEEE International Conference on Consumer Electronics (ICCE) (Berlin: IEEE).

[B42] Rother C. Kolmogorov V. Blake A. (2004). Grabcut: interactive foreground extraction using iterated graph cuts. ACM Trans. Graph. 23, 309–314. 10.1145/1015706.1015720

[B43] Ruben N. E. (2015). Remote Heart Rate Estimation Using Consumer-Grade Cameras [Dissertation]. Logan, UT: Utah State University.

[B44] Rubinstein M. (2013). Analysis and Visualization of Temporal Variations in Video [Dissertation]. Cambridge, MA: Massachusetts Institute of Technology.

[B45] Sahindrakar P. de Haan G. Kirenko I. (2011). Improving Motion Robustness of Contact-Less Monitoring of Heart Rate Using Video Analysis. Eindhoven, The Netherlands: Technische Universiteit Eindhoven, Department of Mathematics and Computer Science.

[B46] Saragih J. M. Lucey S. Cohn J. F. (2011). Deformable model fitting by regularized landmark mean-shift. Int. J. Comput. Vis. 91, 200–215. 10.1007/s11263-010-0380-4

[B47] Soleymani M. Lichtenauer J. Pun T. Pantic M. (2012). A multimodal database for affect recognition and implicit tagging. IEEE Trans. Affect. Comput. 3, 42–55. 10.1109/T-AFFC.2011.25

[B48] Soleymani M. Villaro-Dixon F. Pun T. Chanel G. (2017). Toolbox for emotional feAture extraction from physiological signals (TEAP). Front. ICT 4:1 10.3389/fict.2017.00001

[B49] Starr I. Rawson A. J. Schroeder H. A. Joseph N. R. (1939). Studies on the estimation of cardiac output in man, and of abnormalities in cardiac functions, from the heart’s recoil and the blood’s impacts; the ballistocardiogram. Am. J. Physiol. 127, 1–28.

[B50] Stricker R. Müller S. Gross H. M. (2014). “Non-contact video-based pulse rate measurement on a mobile service robot”, in Robot and Human Interactive Communication, 2014 RO-MAN: The 23rd IEEE International Symposium on (Edinburgh, Scotland: IEEE), p. 1056–1062.

[B51] Sun Y. Hu S. Azorin-Peris V. Kalawsky R. Greenwald S. (2013). Noncontact imaging photoplethysmography to effectively access pulse rate variability. J. Biomed. Opt. 18, 061205–061205. 10.1117/1.JBO.18.6.061205 23111602

[B52] Sun Y. Papin C. Azorin-Peris V. Kalawsky R. Greenwald S. Hu S. (2012). Use of ambient light in remote photoplethysmographic systems: comparison between a high-performance camera and a low-cost webcam. J. Biomed. Opt. 17, 0370051–03700510. 10.1117/1.JBO.17.3.037005 22502577

[B53] Tarassenko L. Villarroel M. Guazzi A. Jorge J. Clifton D. A. Pugh C. (2014). Non-contact video-based vital sign monitoring using ambient light and auto-regressive models. Physiol. Meas. 35, 807. 10.1049/htl.2014.0077 24681430

[B54] Tarvainen M. P. Ranta-Aho P. O. Karjalainen P. A. (2002). An advanced detrending method with application to HRV analysis. IEEE Trans. Biomed. Eng. 49, 172–175. 10.1109/10.979357 12066885

[B55] Tompkins W. J. (1993). Biomedical Digital Signal Processing: C-language Examples and Laboratory Experiments for the IBM PC. Hauptbd. Editorial Prentice Hall.

[B56] Tran D. N. Lee H. Kim C. (2015). “A robust real time system for remote heart rate measurement via camera,” in 2015 IEEE International Conference on Multimedia and Expo (ICME) (Torino, Italy: IEEE).

[B57] Tulyakov S. Alameda-Pineda X. Ricci E. Yin L. Cohn J. F. Sebe N. (2016). “Self-adaptive matrix completion for heart rate estimation from face videos under realistic conditions,” in Proceedings of the IEEE Conference on Computer Vision and Pattern Recognition (Las Vegas, NV: IEEE).

[B58] Verkruysse W. Svaasand L. O. Stuart Nelson J. (2008). Remote plethysmographic imaging using ambient light. Opt. Exp. 16, 21434–21445. 10.1364/OE.16.021434 19104573 PMC2717852

[B59] Vezhnevets V. Sazonov V. Andreeva A. (2003). “A survey on pixel-based skin color detection techniques”, in Proc. Graphicon, Vol. 3, 85–92.

[B60] Villarroel M. Jorge J. Pugh C. Tarassenko L. (2017). “Non-contact vital sign monitoring in the clinic,” in Automatic Face & Gesture Recognition (FG 2017), 2017 12th IEEE International Conference on (IEEE).

[B61] Viola P. Jones M. (2001). “Rapid object detection using a boosted cascade of simple features,” in Computer Vision and Pattern Recognition, 2001. CVPR 2001. Proceedings of the 2001 IEEE Computer Society Conference on, Vol. 1 (Kauai, HI: IEEE).

[B62] Wang W. den Brinker A. C. Stuijk S. de Haan G. (2017). “Color-distortion filtering for remote photoplethysmography,” in Automatic Face & Gesture Recognition (FG 2017), 2017 12th IEEE International Conference on (IEEE).

[B63] Wei L. Tian Y. Wang Y. Ebrahimi T. Huang T. (2012). “Automatic webcam-based human heart rate measurements using laplacian eigenmap,” in Asian Conference on Computer Vision (Berlin, Heidelberg: Springer).

[B64] Werner P. Al-Hamadi A. Walter S. Gruss S. Traue H. C. (2014). “Automatic heart rate estimation from painful faces,” in 2014 IEEE International Conference on Image Processing (ICIP) (Paris, France: IEEE).

[B65] Wold S. Esbensen K. Geladi P. (1987). Principal component analysis. Chemom. Intell. Lab. Syst. 2.1-3, 37–52. 10.1016/0169-7439(87)80084-9

[B66] Wu H. Y. Rubinstein M. Shih E. Guttag J. V. Durand F. Freeman W. (2012). ACM Transactions on Graphics (TOG) – Proceedings of ACM SIGGRAPH 2012, Vol. 31 New York, NY: ACM.

[B67] Xu S. Sun L. Kunde Rohde G. (2014). Robust efficient estimation of heart rate pulse from video. Biomedical Opt. Exp. 5, 1124–1135. 10.1364/BOE.5.001124 24761294 PMC3985994

[B68] Yang F. (2006). 独立分量分析的原理与应用, Tsinghua University Press.

[B69] Yu Y.-P. Kwan B. H. Lim C. L. Wong S. L. Raveendran P. (2013). “Video-based heart rate measurement using short-time Fourier transform,” in Intelligent Signal Processing and Communications Systems (ISPACS), 2013 International Symposium on (Okinawa, Japan: IEEE).

[B70] Yu Y.-P. Raveendran P. Lim C.-L. (2014). “Heart rate estimation from facial images using filter bank,” in Communications, Control and Signal Processing (ISCCSP), 2014 6th International Symposium on (Athens, Greece: IEEE).

[B71] Yu Y.-P. Raveendran P. Lim C.-L. (2015). Dynamic heart rate measurements from video sequences. Biomed. Opt. Exp. 6, 2466–2480. 10.1364/BOE.6.002466 26203374 PMC4505702

[B72] Zaunseder S. Heinke A. Trumpp A. Malberg H. (2014). ““Heart beat detection and analysis from videos.” Electronics and Nanotechnology (ELNANO),” in 2014 IEEE 34th International Conference on (Kyiv, Ukraine: IEEE).

[B73] Zhang Z. Zha H. (2004). Principal manifolds and nonlinear dimensionality reduction via tangent space alignment. SIAM J Sci. Comput. 26, 313–338. 10.1137/S1064827502419154

