# Supplementary material for: A Comparative Survey of Methods for Remote Heart Rate Detection From Frontal Face Videos
Source: Front Bioeng Biotechnol. 2018 May 1;6:33. doi: 10.3389/fbioe.2018.00033 (PMC5938474; doi:10.3389/fbioe.2018.00033)
Supplement: Supplementary file 1 [file Table_1.docx]

Supplementary Material

A Comparison of Methods for Remote Heart Rate Detection From Frontal Face Videos

Chen Wang*, Thierry Pun, Guillaume Chanel

*** Correspondence:** Chen Wang: chen.wang@unige.ch

Appendix I

**Table 1. Experiment Setting (NM = Not Mentioned)**

| Paper | Resolution | Frame Rate (fps) | Color Chanel | Illumination | Camera | Distance | Video length | Still | Move | Ground Truth |
| --- | --- | --- | --- | --- | --- | --- | --- | --- | --- | --- |
| Verkruysse, et al. (2008) | 640x480/ 320x240 | 15/30 | 24bit RGB | Daylight & fluorescent lights/surgery lamps | Canon Powershot models | 1-2m | 30s to several mins | √ | × | Commercial pressure cuff |
|  |  |  |  |  |  |  |  |  |  |  |
| Poh, et al (2010) | 640x480 | 15 | 24bit RGB | Indoor sunlight | Webcam | 0.5m | NM | √ | √ | Finger BVP |
| Poh, et al (2011) | 640x480 | 15 | 24bit RGB | Indoor sunlight | Webcam(iSight) | 0.5m | NM | √ | √ | Finger BVP |
| Pursche, et al. (2012) | 640x480 | 30 | 24bit RGB | Indirect sunlight/office fluorescent lights | Webcam | 0.5m | 2min | NM | NM | Heart Rate Belt |
| Wu, et al. (2012) | 640x480 | 45 | YIQ | NM | NM | NM | NM | √ | × | Hospital-grade monitor |
| Kwon, et al. (2012) | 640x480 | 29.99 | 24bit RGB | Indoor sunlight | Iphone 4 front-facing camera | 0.3m | 1min | √ | × | ECG |
| Wei, et al. (2012) | 640x480 | 30 | 24bit RGB | Indoor sunlight/spot light | Webcam | NM | 30s | √ | slight | finger pulse oximetry |
| Stricker, et al. (2014) | 640x480 | 30 | 24bit RGB | Indoor daylight | eco274CVGE camera | 1.1m | 60s | √ | √ | NM |
| Xu, et al. (2014) | 1280 x 720 / 800 x 480 | 30 /15 | 24bit RGB | indoor and outdoor natural ambient light | Nikon CoolPix L610/Smartphone(Huawei U8652) | NM | 45 -90s | √ | × | Finger pulse oximeter (Santamedical) |
| Balakrishnan, et al. (2013) | 1280x720 | 30 | RGB | Natural, insolated environments | Panasonic Lumix GF2 camera | NM | 70-90s | √ | × | ECG |
| Li, et al. (2014) | 640x480/MAHNOB | 30 / 61 | 24bit RGB | Indoor fluorescent lighting | IPAD frontal iSight camera | 0.35m | 40s | ×/√ | ×/√ | ECG |
|  |  |  |  |  |  |  |  |  |  |  |
| Zaunseder, et al. (2014) | 300x200 | 100 | RGB | NM | UI-5240CP-CHQ IDS | NM | 60s | √ | × | ECG PPG from earlobe |
|  |  |  |  |  |  |  |  |  |  |  |
| De Haan, et al. (2013) | 1024x752 | 20 | 24bit RGB | Profession studio illumination | type USB UI-2230SE-C of IDS Gmbh | NM | 1 min | √ | √ | Finger pulse oximeter |
| Chen, et al. (2015) | NM | 30 | RGB | Indoor | Panasonic SMC-FX07GT | 7-9cm | 30s | √ | × | sphygmomanometer |
| Chen, et al. (2017) | 1920x1080 | 24 | 24bit RGB | ambient light | Intel RealSense Camera VF0800 | 0.5-1.0m | 30s | NM | NM | Finger BVP |
| Villarroel, et al. (2017) | NM | 12 | RGB | NM | 5 megapixel camera | around 1.0m | 4 hours | × | √ | pulse oximeter & ECG |
| McDuff, et al. (2017) | 658×492 | 120 | RGB | full color spectrum bulbs with frost diffuser | Scout scA640-120gc | NM | 1 hour | √ | √ | ECG |
| Wang, et al. (2017) | NM | NM | RGB | sun light and fluorescent lighting | Scout scA640-120gc | 2 m | 1 hour | √ | √ | ECG |
| Tarassenko, et al. (2014) | NM | 12 | RGB | NM | 5 megapixel camera | around 1.0m | 4 hours | × | √ | pulse oximeter & ECG |
| Tulyakov, et al. (2016) | MAHNOB-HCI & MMSE-HR | 61 fps | RGB | Indoor | industrial camera | NM | 30s | × | √ | ECG |
| Monkaresi, et al. (2014) | 640x480 | 30 fps | 24bit RGB | Indoor fluorescent light & sunlight | Webcam(Logitech Webcam Pro 9000) | NM | NM | √ | √ | ECG GSR |
| Osman, et al. (2015) | 640x480 | 22-30 fps | 24bit RGB | NM | Macbook Pro webcam | NM | 12.5mins | × | √ | Finger BVP |
| Lewandowska, et al. (2011) | 640x480 | 20 fps | RGB | Indoor sunlight | Logitech Webcam 9000 Pro | NM | 30s | √ | c | ECG |
| Wei, et al. (2012) | 640x480 | 30 fps | 24bit RGB | Indoor Sunlight/spot light | Webcam(Logitech C170) | NM | 30s | √ | √ | Finger pulse oximeter |
| Xu, et al. (2014) | 1280x720/ 800x480/ 1280x720 | 30 fps/15 fps/30 fps | 24bit RGB | Indoor/ Outdoor ambient light | Nikon CoolPix L610/ Huawei U8652/Iphone4 | NM | 45-90s | NM | NM | Finger pulse oximeter |
| Sahindrakar, et al. (2011) | 640x480 | 20 fps | 24bit RGB | Indoor | uEye video camera | 1.5m | NM | √ | √ | ECG |
| Tran, et al. (2015) | 640x480 | 10 fps | 24bit RGB | Indoor | Logitech C920 webcam | 0.5-1.2m | 66s | √ | √ | Finger pulse oximeter |
| Werner, et al. (2014) | BioVid Heat Pain Database | 25 fps | RGB |  |  |  | 8s | √ | √ | ECG |
| Sun, et al. (2013) | 256x384 | 200 fps | monochrome | Indoor | CMOS camera | 400 mm | 4mins |  |  | Figure pulse oximeter |
| Mestha, et al. (2014) | 640x480 | 20 fps | 24bit RGB | Lab room TL light/daylight | uEye video camera | 1.5m | NM | √ | √ | ECG |
| Datcu, et al. (2013) | 252x350 | 242 fps | RGB | Indoor fluorescent lamp | Pike high speed camera | NM | 30s | √ | × | Pulse measurement(Mobi 8) |
| Kumar, et al. (2015) | 1280x1024 | 30 fps | Monochrome/ 24bit RGB | Fluorescent lighting | Monochrome /color camera (Flea3) | 0.5m | 40s | √ | √ | Pulse oximeter PPG |
| Irani, et al., (2014) | 640x480 | 30 fps | RGB | Indoor | Logitech webcam | NM | NM | √ | √ | ECG |
| Jensen, et al. (2014) | 640x480 | 30 fps | 24bit RGB | outdoor | Webcam (Lenovo X220) | NM | 1s | √ | × | PPG |
| Yu, et al. (2015) | 1440x1080 | 25 fps | 24bit RGB | NM | Sony HDR-PJ580V | 0.6m | 3-8s | √ | × | Polar chest strap |
|  |  |  |  |  |  |  |  |  |  |  |
| Lam, et al. (2015) | MAHNOB HCI | 61 fps | 24bit RGB | indoor | industrial camera | NM | NM | × | √ | ECG |
|  |  |  |  |  |  |  |  |  |  |  |
| Moreno, et al. (2015) | 640x480 | 30 fps | RGB | 40W lamp | Compact camera(Canon lxus 80is) | 0.8m | 5 mins | √ | × | Polar chest strap |
